# Biotin IgM Antibodies in Human Blood: A Previously Unknown Factor Eliciting False Results in Biotinylation-Based Immunoassays

**DOI:** 10.1371/journal.pone.0042376

**Published:** 2012-08-03

**Authors:** Tingting Chen, Lea Hedman, Petri S. Mattila, Laura Jartti, Tuomas Jartti, Olli Ruuskanen, Maria Söderlund-Venermo, Klaus Hedman

**Affiliations:** 1 Department of Virology, Haartman Institute, University of Helsinki, Helsinki, Finland; 2 Department of Otorhinolaryngology, University of Helsinki and Helsinki University Central Hospital, Helsinki, Finland; 3 Department of Geriatrics, Turku City Hospital, Turku, Finland; 4 Department of Pediatrics, Turku University Hospital, Turku, Finland; 5 Department of Virology and Immunology, Helsinki University Central Hospital Laboratory Division, Helsinki, Finland; INSERM U1094, University of Limoges School of Medicine, France

## Abstract

Biotin is an essential vitamin that binds streptavidin or avidin with high affinity and specificity. As biotin is a small molecule that can be linked to proteins without affecting their biological activity, biotinylation is applied widely in biochemical assays. In our laboratory, IgM enzyme immuno assays (EIAs) of µ-capture format have been set up against many viruses, using as antigen biotinylated virus like particles (VLPs) detected by horseradish peroxidase-conjugated streptavidin. We recently encountered one serum sample reacting with the biotinylated VLP but not with the unbiotinylated one, suggesting in human sera the occurrence of biotin-reactive antibodies. In the present study, we search the general population (612 serum samples from adults and 678 from children) for IgM antibodies reactive with biotin and develop an indirect EIA for quantification of their levels and assessment of their seroprevalence. These IgM antibodies were present in 3% adults regardless of age, but were rarely found in children. The adverse effects of the biotin IgM on biotinylation-based immunoassays were assessed, including four inhouse and one commercial virus IgM EIAs, showing that biotin IgM do cause false positivities. The biotin can not bind IgM and streptavidin or avidin simultaneously, suggesting that these biotin-interactive compounds compete for the common binding site. In competitive inhibition assays, the affinities of biotin IgM antibodies ranged from 2.1×10^−3^ to 1.7×10^−4 ^mol/L. This is the first report on biotin antibodies found in humans, providing new information on biotinylation-based immunoassays as well as new insights into the biomedical effects of vitamins.

## Introduction

Biotin, also known as vitamin B7 and vitamin H, is a water-soluble micronutrient required by most organisms. In mammals, biotin acts as a coenzyme for four carboxylases: propionyl-coenzyme A (CoA) carboxylase, pyruvate carboxylase, methylcrotonoyl-CoA carboxylase, and acetyl-CoA carboxylase. The first three are located in mitochondria and the fourth in the cytoplasm. These enzymes catalyze critical reactions in the intermediary metabolism of gluconeogenesis, fatty acid synthesis, and amino acid catabolism [1], [2]. Therefore, insufficient biotin intake may lead to a number of clinical abnormalities, including hair loss, dermal rash, growth retardation, neurological disorders and a higher vulnerability to infections [1], [3]–[5].

Biotin deficiency is rare among constitutionally healthy people as this nutrient is widely distributed in foods and also amply synthesized by the intestinal flora, meeting with our low daily requirement (approximately15–70 µg/day) [1], [2]. However, biotin deficiency can ensue upon excessive consumption of raw eggs, due to its constituent, avidin, binding biotin at high affinity in the alimentary tract, and preventing its intestinal absorption [3], [6]–[8]. In the human body, only free biotin can function in metabolism, whereas most of the dietary biotin is protein-bound [2]. Biotinidase, a hepatic enzyme synthesized in the liver and secreted into the blood, is responsible for processing protein-bound biotin and recycling biotin [5], [9]. In human serum, biotin circulates in free form or protein-bound, for uptake by cells and tissues including liver cells, cerebral capillaries, basolateral membrane vesicles of placenta, and peripheral blood mononuclear cells [1], [2].

**Figure 1:**
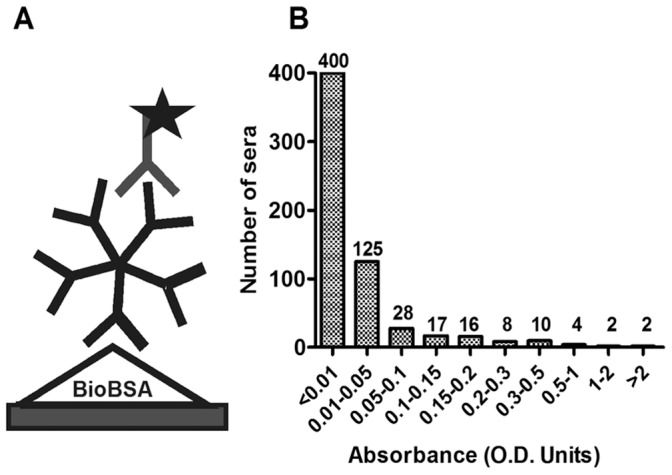
Biotin IgM indirect EIA. (A) Layout of biotin IgM indirect EIA (Bio-EIA). (B) The distribution of EIA absorbance values of 612 adults’ serum samples of Group 1 and Group 2. Five-pointed star: HRP conjugates.

Due to its small size, biotin can be covalently linked to a number of proteins without affecting their biological activity. As biotin binds avidin or streptavidin with extremely high affinity and specificity, biotinylation of proteins and macromolecules is applied widely in biochemical assays [10]–[14]. In our laboratory, for detecting IgM antibodies against many different viruses, µ-capture enzyme immuno assays (EIAs) have been set up by using as antigen biotinylated virus like particles (VLPs) detected by horseradish peroxidase (HRP)-conjugated streptavidin [15]–[18]. As the presence of pathogen-specific IgM antibody is applied as a marker of ongoing or recent infection, the specificity of the assay is critically important. We recently, aiming at employing as a negative control in our Merkel cell polyomavirus (MCV) IgM serology, and encountering strong reactivity in the serum of one of us authors, found the IgM to react with the biotinylated VLPs but not with the unbiotinylated ones. Prompted by this preliminary finding, the following study was initiated.

**Figure 2:**
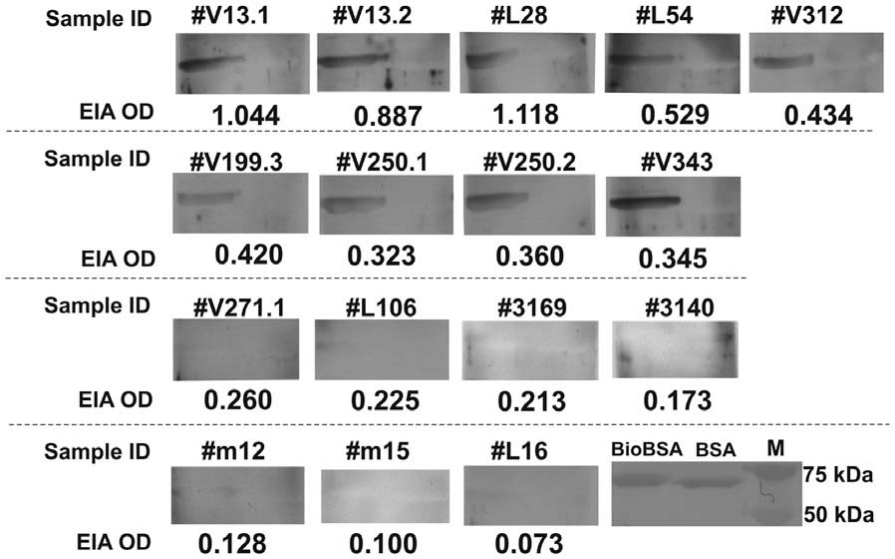
Correspondence between EIA absorbances and western blot reactivities. In each blot the left lane contains biotinylated BSA, and the right lane contains nonbiotinylated BSA. In lower right corner is the blot treated with the protein stain Ponceau S red.

In this study, we looked for human serum IgM antibodies reacting with biotin (Bio-IgM) and developed an indirect EIA to quantify their level and assess their seroprevalence. The adverse effects of biotin IgM on biotinylation-based immunoassays were assessed, including four inhouse virus IgM EIAs and one commercial assay. The biotin IgM and streptavidin/avidin were shown to compete for binding to biotin. The affinities of the biotin IgM were also determined.

**Table 1:** Biotin IgM seroprevalence in the general population.

Cohort	Subjects no.	Positive subjects no.	Seropositivity
Adults	144	4	2.8%
Seniors	316	10	3.2%
Children	349	1	0.3%

## Results

### Detection of Biotin Antibodies in Human Sera and Assessment of Seroprevalence among Adults and Children

To measure seroreactivities against biotin among the general population, an indirect IgM EIA (Bio-EIA) was set up with biotinylated BSA as antigen. The EIA OD values of 612 serum samples from 459 adults were plotted in a bar chart (Figure 1): 553 (90%) samples showed values <0.1 OD units, whereas 26 (4.2%) showed values >0.2. To confirm the EIA results, western blot (WB) was employed, as shown in Figure 2 with representative samples from each patient group. Positive WB signals were uniformly observed among the samples with EIA reactivities ≥0.3 OD units, but were rare among those with reactivities below 0.3. No signal against unbiotinylated BSA was seen with any of the samples.

**Figure 3:**
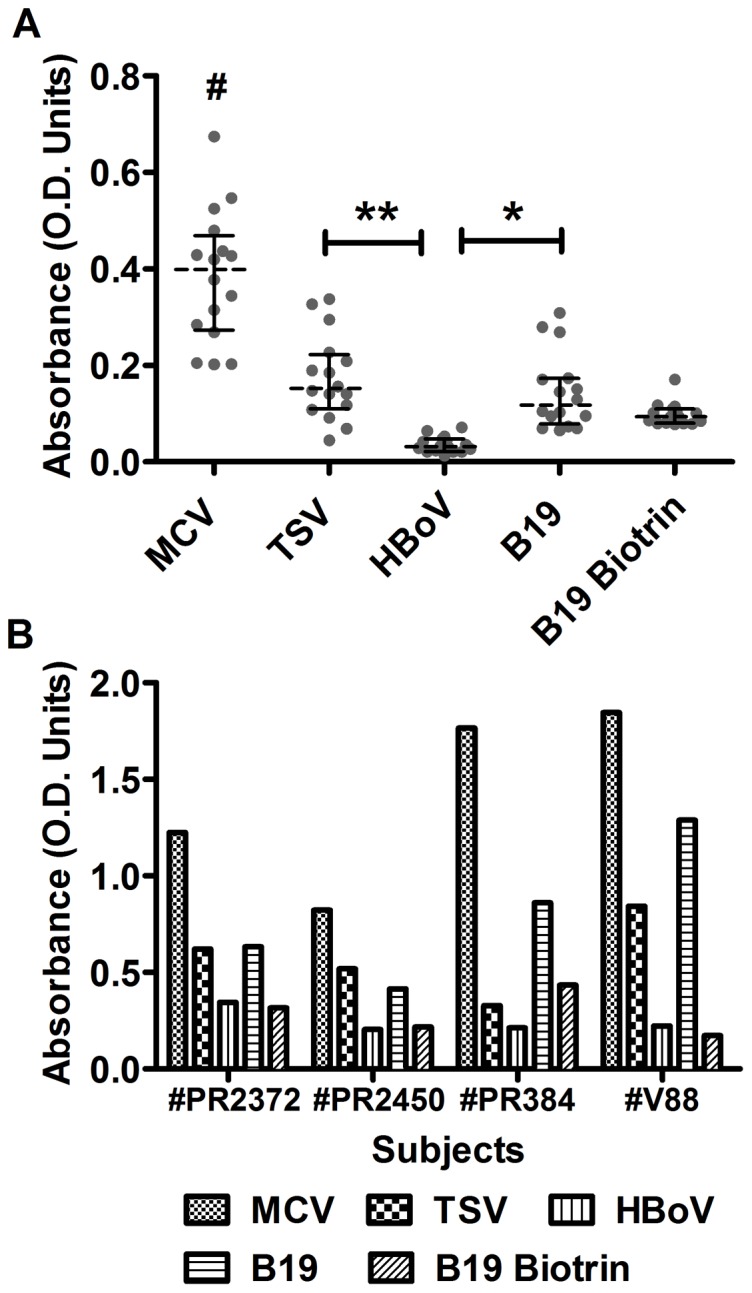
Effect of biotin IgM on biotinylation-based virus IgM EIAs. (A) The distribution of reactivities measured by four inhouse virus IgM EIAs and one commercial kit; serum samples containing moderate or low levels of biotin IgM (EIA absorbance <1.200). The dotted lines indicate median absorbance values, and bars represent interquartile values. Absorbance values were analyzed using one-way ANOVA for overall comparison (P<0.0001), followed by Tukey-Kramer HSD test for pairwise comparison. **#**P<0.0001 compared to all the others; **P<0.0001, *P<0.05. (B) The reactivities with four serum samples containing high levels of biotin IgM (EIA absorbance >3.000). The cutoff values for positivity and negativity are: MCV 0.260 and 0.207, TSV 0.240 and 0.194, HBoV 0.167 and 0.136, B19 0.220 and 0.171, B19 Biotrin 0.342 and 0.279.

Among the 144 adults comprising students and staff, 4 (2.8%) had biotin IgM and among 316 senior individuals, 10 (3.2%), as shown in Table 1. From four of the latter, 1–3 week follow-up sera were available, and all maintained the biotin IgM signal at invariant intensity. However, the institute staff member who was initially positive with OD 2.5 in Bio-EIA lost his IgM during follow-up of six months, without IgG seroconversion. Among 678 samples from 349 children aged 1–12 years (median 2.5; mean 3.2), only one serum sample was positive. Overall, based on our assays, the seroprevalence of biotin IgM among adults of all ages was 3.0%, and was substantially lower (0.3%) among children. Of note, none of the Bio-IgM-positive samples contained IgG antibodies against biotin; neither did any of the 110 Bio-IgM-negative samples analyzed (data not shown).

**Figure 4:**
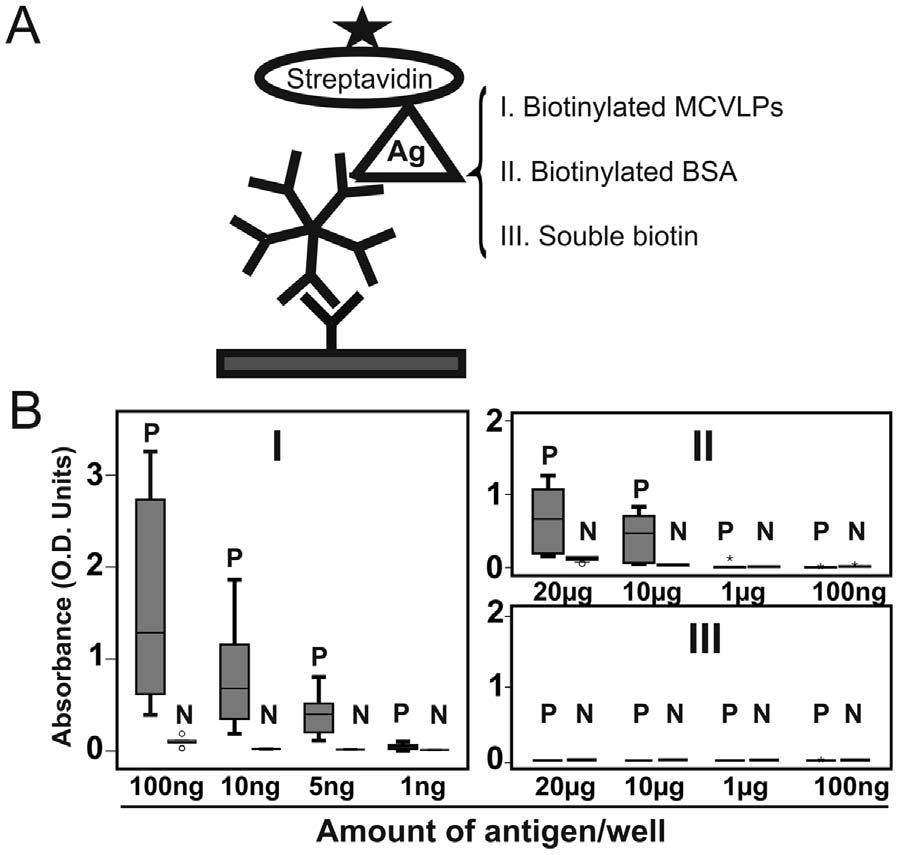
Interaction of biotin IgM with biotin-containing antigens in µ-capture IgM EIAs. Serum samples containing or lacking biotin IgM were applied in µ-capture IgM EIAs including (I) Biotinylated MCVLPs (at concentrations of 100 ng, 10 ng, 5 ng, 1 ng/100 µL), (II) Biotinylated BSA (at concentrations of 20 µg, 10 µg, 1 µg, 100 ng/100 µL) or (III) Soluble biotin (at concentrations of 20 µg, 10 µg, 1 µg, 100 ng/100 µL). The reactivities were plotted in boxplot. Five-pointed star: HRP conjugates. **P**: positive group comprising nine Bio-IgM positive sera. **N**: negative group comprising nine Bio-IgM-negative sera.

**Figure 5:**
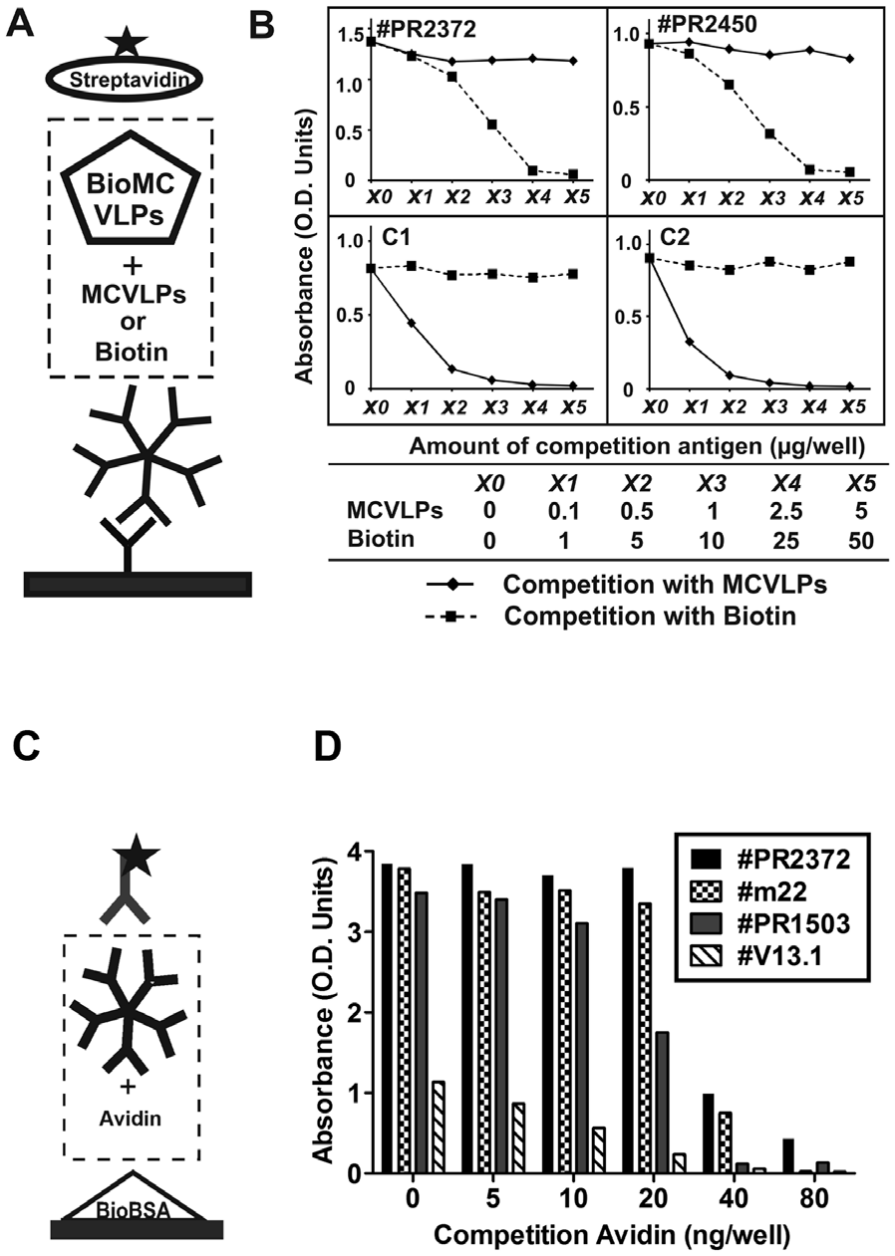
Competition assays. (A) In inhouse MCV IgM EIA, biotinylated MCV VLPs (10 ng/100 µL) were mixed with increasing concentrations of either unbiotinylated MCV VLPs or soluble biotin and added onto the µ-capture plates (100 µL/well). (B) The upper figures show two serum samples positive for biotin IgM; the lower figures show two serum samples positive for MCV IgM. **(C and D)** In Bio-EIA, four samples positive for biotin IgM were coincubated with avidin and applied to the assay. Five-pointed star: HRP conjugates.

### Assessment of the Biotin Antibody Effect on IgM Antibody Assays

To assess whether the biotin IgM affects biotinylation-based antiviral IgM assays, all available Bio-IgM-positive serum samples were examined by four inhouse virus IgM EIAs of µ-capture format [MCV, *Trichodysplasia-spinulosa* polyomavirus (TSV), human bocavirus 1 (HBoV), parvovirus B19 (B19V)] and by one commercial kit (B19V EIA of Biotrin). In Figure 3A, 16 adults’ sera with Bio-EIA ODs between 0.312 and 1.118, showed in inhouse-EIA ODs of 0.202–0.674 for MCV, of 0.045–0.338 for TSV, of 0.013–0.072 for HBoV and of 0.066–0.309 for B19V, and ODs of 0.078–0.171 in the commercial B19V EIA. In contrast, four samples (one senior sample and three of the four B19V-IgM false positive samples) with Bio-EIA reactivities exceeding OD 3, showed elevated reactivities in all assays (Figure 3B). With each of these sera, the ODs in MCV EIA exceeded those in the other EIAs. According to the cutoff values for each assay, among those 20 serum samples, the numbers of respective samples that were positive, negative, and at borderline range in each assay were 17, 3, 0 for MCV, 7, 11, 2 for TSV, 4, 16, 0 for HBoV, 7, 12, 1 for B19V (inhouse), and 1, 18, 1 for B19V (Biotrin).

**Figure 6:**
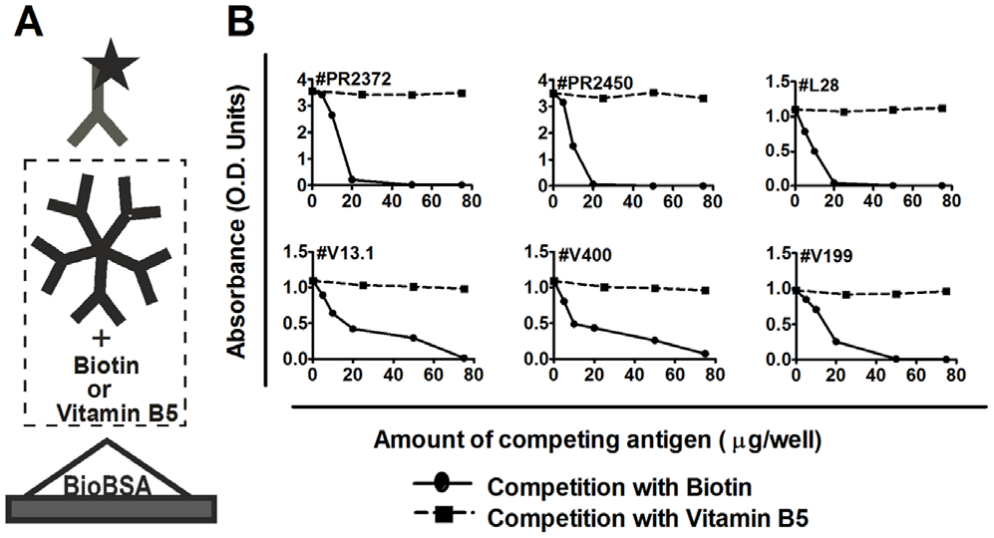
Competition assays on Bio-IgM-positive serum samples. In Bio-EIA, sera preincubated with increasing concentrations of either soluble biotin (5, 10, 20, 50, 75 µg/100 µL) or vitamin B5 (25, 50, 75 µg/100 µL) for 1 hr at RT, and applied into the plates (100 µL/well). The reactivities declined to undetectable levels upon serum preincubation with biotin, but not with vitamin B5.

**Figure 7:**
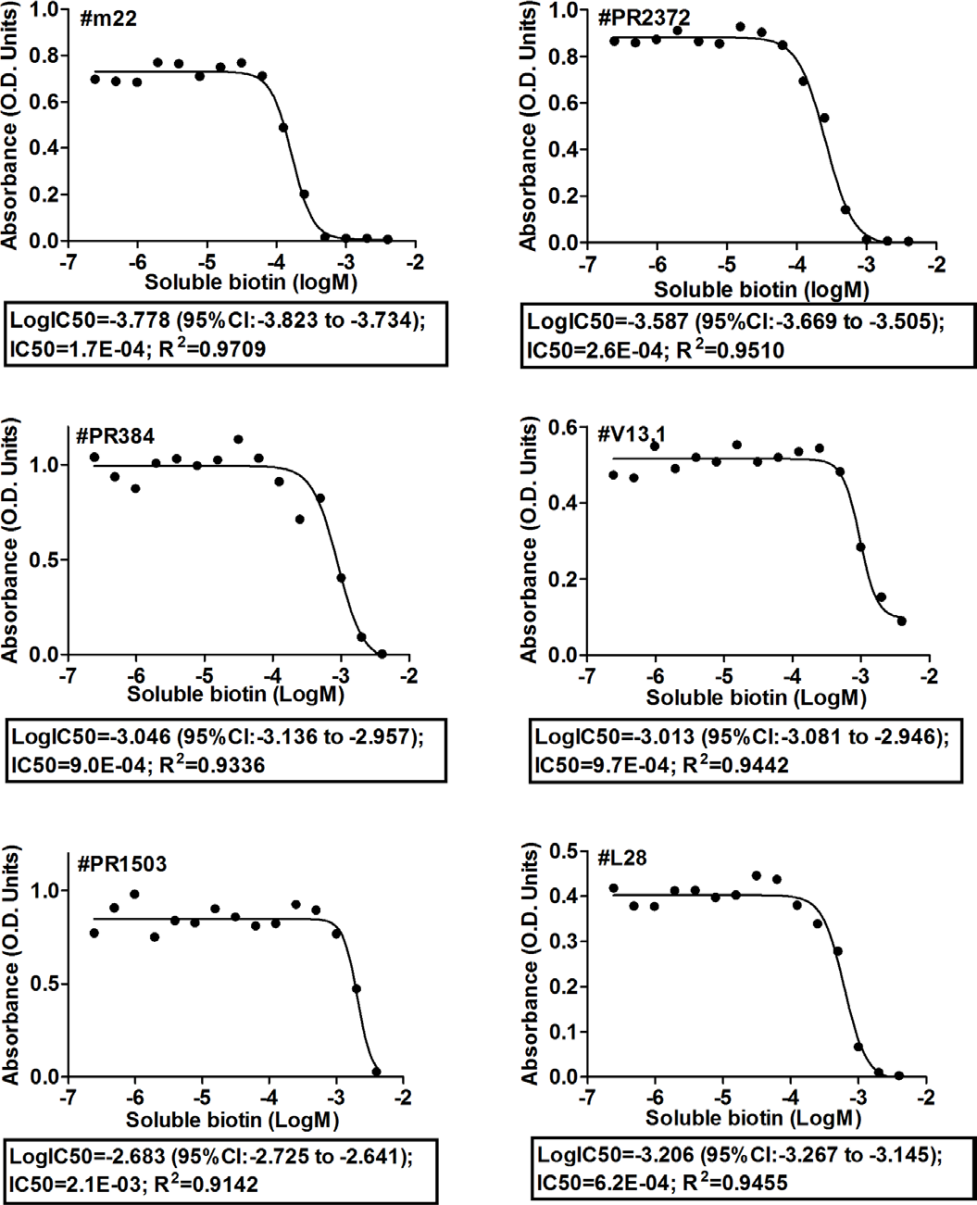
Affinity measurement. Diluted sera were incubated with increasing concentrations of biotin over night, and then transferred into Bio-EIA plates.The amount of antibody bound to biotinylated BSA in the solid phase was measured.

### Competition between IgM and Streptavidin/avidin for Binding to Biotin

Serum samples, 9 Bio-IgM positive and 9 negative, were applied in a µ-capture EIA (inhouse EIA, Figure 4), and therein tested with one of three different antigens: biotinylated MCV virus like particles (MCVLPs), biotinylated BSA and soluble biotin. In Figure 4B-I, the Bio-IgM-positive samples showed positive IgM results with biotinylated MCVLPs at a minimum amount of 5 ng/well, with a dose-dependent increase in reactivity. Correspondingly, they also yielded positive IgM results (Figure 4B-II) with biotinylated BSA at 10 µg/well or higher, but negative results with soluble biotin regardless of concentration (Figure 4B-III). By comparison, none of the Bio-IgM-negative sera showed any reactivity using as antigen biotinylated MCVLPs, biotinylated BSA or souble biotin. Competition experiments (Figure 5A and B) were performed with samples reactive with biotinylated MCVLPs in a µ-capture IgM EIA (MCV inhouse EIA). With two Bio-IgM-positive sera, the reactivities were blocked dose-dependently by increasing concentrations of soluble biotin, but not by non-biotinylated MCVLPs. Correspondingly, with two MCV-IgM-positive sera, the reactivities were blocked by MCVLPs, but not by biotin. Furthermore, as shown in Figure 5C and D, the Bio-EIA reactivities of positive sera were efficiently abolished by coincubation with avidin dose-dependently with increasing concentrations.

### Affinity Determination (Half-maximal Inhibitory Concentration IC50)

The binding specificity and affinity of biotin IgM antibodies were further studied by competition experiments (Figure 6A), in which the binding of a given Bio-IgM to solid-phase biotinylated BSA was measured in the presence of homologous soluble biotin and heterologous vitamin B5 (Pantothenic acid, Sigma-Aldrich, P2250). In Figure 6B, the reactivities of six serum samples were inhibited by biotin, but not by vitamin B5. In Figure 7, with six serum samples diluted, the binding of biotin IgM to biotinylated BSA was inhibited in a dose-dependent fashion, with IC50s ranging from 2.1×10^−3^ to 1.7×10^−4^ mol/L.

## Discussion

We report for the first time the occurrence of IgM antibodies to biotin in humans of the general population. The biotin IgM shown here interacts non-covalently with biotin, differently from previously reported biotin-binding immunoglobulins (BBI), which were formed by biotin covalently bound to the constant region of IgM or IgG [19], [20]. For seroprevalence determination, we developed an EIA for these antibodies. The cutoff for positivity in our assay was set according to immunoblot results, whereby we found a seroprevalence of 3% among Nordic adults. Existing data on occurrence of human antibodies to vitamins are scarce except for anti-vitamin D IgG antibodies that have been observed at a low prevalence in patients with autoimmune diseases [21]. As autoantibodies do occur also in healthy people, e.g. rheumatoid factor (RF) in 1–3% of the general population [22]–[24], the possibility that the biotin IgM has an association with autoimmunity deserves to be explored in the future. On the other hand, with an essential role in metabolism, biotin can exist in the human body in various forms, such as biotinylated carboxylases and metabolites [1]. Another interesting possibility stems from the fact that biotin only accounts for half of the total avidin-binding substances in human plasma, as biotin metabolites that originate from cellular oxidation account for the rest [25], [26]. As those metabolites in molecular structure resemble biotin [1], it is possible that the biotin IgM is derived from an immune response to these metabolites, possibly due to an imbalance in biotin metabolism.

Since biotin IgM was initially found in a serum sample with false positivity in our inhouse IgM assay, we determined the susceptibility of several assays to it. We found that, serum samples with high levels of biotin IgM affected adversely all virus IgM assays, while samples containing low levels of the antibody showed low interference level in most assays. Indeed, biotin IgM was shown to be an important contributor to false positivities in virus IgM assays. Moreover, our results showed that among µ-capture IgM assays for four different viruses, the effects of biotin IgM were different. The MCV IgM EIA was more susceptible than the others. This difference may depend on the conformation of viral particles or their degree of biotinylation. Thus, at least two ways emerge to limit the effect of the biotin IgM: one is to optimize the amount of biotin in VLP trimming, and the other is to employ an appropriate borderline zone upon setting up the cutoffs according to the false reactivities derived from biotin IgM.

We did study in detail whether the different effects of biotin antibodies on IgM assays would relate with the conformations of the biotinylated antigens. As MCV VLPs were more susceptible than BSA, it is possible that ordered supramolecular assemblies are more susceptible than isolated polypeptides. Of note, biotin itself in solution never gave a positive signal. To account for this, three possibilities existed: (a) the soluble biotin did not bind to the biotin IgM captured on the plate; (b) the IgM-attachment abolished biotin’s ability to bind to streptavidin; (c) IgM would bind the long spacer arm (LC) of biotinylation reagent (Sulfo-NHS-LC-Biotin) instead of the biotin itself. To find the answer, we performed competition assays with soluble biotin in MCV IgM EIA. The fact that soluble biotin displaced biotinylated MCV VLPs without causing positive results itself, proved that biotin could not bind IgM antibodies and streptavidin simultaneously and that the binding was specific for the biotin itself and not the spacer arm. As avidin is a natural constituent in human’s diet and binds to biotin similarly as streptavidin does [12], we performed competition assays with avidin, which confirmed that IgM and streptavidin/avidin compete with each other for binding to biotin. As biotin is a small molecule (244Da), IgM and streptavidin/avidin probably share on biotin the same binding site. On the other hand, as avidin can prevent the intestinal absorption of biotin [3], [7], [8], it is tempting to speculate a similar function for the biotin IgM in blood. Indeed, whether the biotin IgM can prevent biotin uptake by tissues or cells is an exciting topic for further study.

We furthermore assessed the binding strength of biotin IgM by measuring IC50 [27]. The observed affinity level of 10^−3^–10^−4^ mol/L is similar to that of many natural antibodies with their respective antigens [28]–[32]. An interesting possibility is that the biotin IgM belongs to the group of germ line-encoded immunoglobulins generated without known antigenic stimulation. Most natural antibodies do belong to the IgM class and are presumed to be driven by endogenous host antigen [28], [30], [33]. As biotin has the ability to interact with host proteins [1], [2], [34], the resulting complexes might drive the production of these antibodies toward the ligand. However, the biotin IgM seemed specific, as it in our study did not react with vitamin B5, a vitamin so closely related to biotin that it can inhibit the intestinal absorption of the latter [35].

Since our study cohorts are residents in Finland, it would be worthwhile to investigate biotin antibodies in non-Finnish populations. On the other hand, as our seroprevalence data are not population-based, they do not necessarily disclose the precise prevalence of biotin IgM antibodies even among the Finnish population. Nevertheless, the finding of biotin IgM in human sera opens a new venue for research of vitamin-directed immunity. The potential effects of these antibodies on human health call for investigation. These antibodies might adversely affect the uptake of biotin from circulation, or might help in clearance of excessive biotin and its metabolites in blood.

## Materials and Methods

### Ethics Statement

The study protocol was reviewed and approved by the Ethics Committees of Turku and Helsinki University Hospitals. Written informed consent was obtained from all participants. If senior participant was unsure about the consent, or he/she was assessed by a geriatrician to have reduced capacity to consent, a written consent was obtained from the next of kin. If participants were children, consent was obtained from parents or guardians.

### Samples

The occurrence of biotin IgM antibodies was determined in 612 serum samples from two groups of adults and 678 samples from children. The first group, referred to as “Adults” comprised 144 sera from University students and our Institute staff, and the second group, “Seniors”, comprised 468 sera from 316 senior citizens (mean age 82 years, range 70–100). All senior citizens were in-patients of Turku City Hospital and participated to Virel study which aims to investigate detailed molecular virus etiology in the elderly and its links to illness severity and long-term outcome. The last group, “Children”, comprised sera from 349 children at sampling age of 1–12 years (median 2.5; mean 3.2), which were previously studied for MCV and TSV primary infection [36], [37]. In addition, four serum samples observed to cause false reactivities in our inhouse assay for B19V IgM due to biotin antibodies, were also included in the present study but omitted from the calculation of seroprevalence.

### Biotin IgM Indirect Enzyme Immunoassay

An indirect EIA was set up for detection of biotin IgM in human serum samples (Bio-EIA, Figure 1A). For use as antigen, bovine serum albumin (BSA) (Sigma-Aldrich, A7906) was biotinylated by using the EZ-Link Sulfo-NHS-LC-Biotinylation kit (Pierce, Rockford, IL.) in accordance with the manufacturer’s instructions, and dialyzed against PBS. Strips of microtiter wells were placed in lockwell frames (Nunc-Immuno™ Modules and Frames), and coated with 200 ng/well of biotinylated BSA in PBS for 2.5 hours at 37°C. After 5 washes with PBS containing 0.05% Tween-20 (PBST), the wells were blocked in 5% BSA in PBS for 30 min at 37°C, followed by a rinse. The serum samples diluted 1∶100 in PBST were applied in duplicate (100 µl/well) for 60 min at room temperature in a rocking (400 rpm) EIA incubator. After washes with PBST, polyclonal rabbit anti-human IgM-horseradish peroxidase conjugate (DakoCytomation, Glostrup, Denmark) diluted 1∶1,000 in 3% BSA/PBST, was added to each well (100 µl), and incubated for 60 min. After washes, orthophenylene diamine and H_2_O_2_ were added, the reaction was stopped after 25 min with 0.5 M H_2_SO_4_, and the absorbances at 492 nm were recorded. For background reactivity, a BSA-EIA was set up by coating the plates with unbiotinylated BSA and performed as above.

In cutoff determination, all sera yielding OD <0.15 units in Bio-EIA were considered negative, and the samples with higher OD were re-examined by both Bio-EIA and BSA-EIA; and the net OD was obtained by subtraction of BSA-EIA background. Samples with net OD ≥0.3 were considered positive, those with net OD <0.2 were considered negative, and those with net OD between 0.2 and 0.3 were considered borderline.

### Western Blot Analysis

For detection of biotin IgM by immunoblot, 1 µg/lane of biotinylated BSA (Bio-BSA) or BSA was separated by 10% SDS-PAGE and transferred onto nitrocellulose membranes (Whatman Protran BA 85), blocked in 5% BSA in Tris buffered saline containing 0.1% Tween 20 (TBST) for 30 min at room temperature (RT). The serum samples diluted 1∶100 or 1∶50 in 5% BSA/TBST were kept on the blots at 4°C overnight. After washing with TBST, a polyclonal rabbit peroxidase-conjugated anti-human IgM (DakoCytomation, Glostrup, Denmark) diluted 1∶20,000 in 5% BSA/TBST was applied for 1 hour at RT. After washes, the blots were developed using the SuperSignal West Dura Extended Substrate Detection kit (Pierce, Rockford, IL.) and exposed on X-ray films (Fuji Super RX).

### IgM Enzyme Immunoassay

µ-capture EIAs were performed in analogy to our many antiviral IgM EIAs (inhouse EIAs) [15]–[18]. Briefly, serum samples diluted 1∶200 in PBST were applied in duplicate into wells of plates coated with goat anti-human IgM (Cappel/ICN Biomedicals) for 60 min at room temperature. After washes, the biotinylated antigens were applied and incubated for 45 min at 37°C. Then the plates were treated with horseradish peroxidase-conjugated streptavidin (Dako, Glostrup, Denmark) at 1∶12,000 in PBST containing 0.5% BSA for 45 min at 37°C, followed by orthophenylene diamine and H_2_O_2_ for 15 min at 37°C. In competition assay, the biotinylated antigen was mixed with a nonbiotinylated one, and added into the plates (Figure 5A).

### Affinity Determination

Based on the Bio-EIA, a competition assay was set up to measure biotin IgM affinity [27], [29], [38], [39]. Briefly, the sera were diluted for an OD of 0.4 to 1.2 and incubated with increasing concentrations of soluble biotin (Sigma-Aldrich, B4501) over night, to reach equilibrium. The mixtures were then transferred into EIA plates in five replicates and the amount of antibody bound to the solid phase was measured as described above. The mean values were plotted in a graph, yielding a fitting curve by a nonlinear regression program (GraphPad Prism). The IC50 was then calculated from the curve, corresponding to affinity [27], [39].
